# Impact of Neuro-Psychological Factors on Smoking-Associated Lung Cancer

**DOI:** 10.3390/cancers6010580

**Published:** 2014-03-13

**Authors:** Hildegard M. Schuller

**Affiliations:** Experimental Oncology Laboratory, Department of Biomedical and Diagnostic Sciences, College of Veterinary Medicine, University of Tennessee, 2407 River Drive, Knoxville, TN 37996, USA; E-Mail: hmsch@utk.edu; Tel.: +1-865-974-8217; Fax: +1-865-974-5616

**Keywords:** smoking, non-small cell lung cancer, nicotinic receptors, β-adrenergic receptors, sympathicus hyperactivity, psychological stress

## Abstract

Smoking has been extensively documented as a risk factor for all histological types of lung cancer and tobacco-specific nitrosamines and polycyclic aromatic hydrocarbons reproducibly cause lung cancer in laboratory rodents. However, the most common lung cancer, non-small cell lung cancer (NSCLC), frequently develops in never smokers and is particularly common in women and African Americans, suggesting that factors unrelated to smoking significantly impact this cancer. Recent experimental investigations *in vitro* and in animal models have shown that chronic psychological stress and the associated hyperactive signaling of stress neurotransmitters via β-adrenergic receptors significantly promote the growth and metastatic potential of NSCLC. These responses were caused by modulation in the expression and sensitization state of nicotinic acetylcholine receptors (nAChRs) that regulate the production of stress neurotransmitters and the inhibitory neurotransmitter γ-aminobutyric acid (GABA). Similar changes in nAChR-mediated neurotransmitter production were identified as the cause of NSCLC stimulation *in vitro* and in xenograft models by chronic nicotine. Collectively, these data suggest that hyperactivity of the sympathetic branch of the autonomic nervous system caused by chronic psychological stress or chronic exposure to nicotinic agonists in cigarette smoke significantly contribute to the development and progression of NSCLC. A recent clinical study that reported improved survival outcomes with the incidental use of β-blockers among patients with NSCLC supports this interpretation.

## 1. Introduction

Lung cancer is globally the leading cause of cancer deaths, with non-small cell lung carcinoma (NSCLC) predominating [1]. Since the first Surgeon General’s report on the association of smoking with lung cancer in 1964, tobacco control measures have significantly reduced the number of smokers in the US, saving an estimated 8 million lives until today [2]. However, contrary to expectations, the significant decrease in smokers observed over the past five decades has not yielded a correlating decrease in incidence and mortality of NSCLC [3]. Instead, a shift in the incidences of histological subtypes of NSCLC has been observed, with previously leading squamous cell carcinoma declining and adenocarcinoma rising, especially in never smokers, and accounting for about 80% of NSCLC cases in never smokers today [3,4,5]. Lung cancer in never smokers is disproportionately more common in women than men and the majority of lung cancers in never smokers are adenocarcinomas [6]. In addition, African Americans have a higher incidence of lung cancer than the general population, irrespective of smoking history [7]. Collectively, these findings strongly suggest that factors other than smoking play significant roles in the development of lung cancer, particularly adenocarcinoma.

Epidemiology is an imprecise science in that individuals are always exposed to a multitude of factors not controlled by the investigator in addition to those under analysis. This problem is overcome by *in vitro* studies and animal experiments under tightly controlled conditions that have been used for many years to study the mechanisms of cancer initiation, progression and responsiveness to prevention and therapy. Such experimental studies provide the basis for the development of effective cancer preventive and therapeutic strategies. In accord with early findings that nicotine-derived carcinogenic nitrosamines [8] and polycyclic aromatic hydrocarbons [9] contained in cigarette smoke cause lung cancer in laboratory rodents, traditional experimental lung cancer research has primarily focused on the metabolic activation of these chemicals in susceptible tissues and the interaction of reactive metabolites with DNA [10,11]. However, discoveries that nicotine-derived carcinogenic nitrosamines are high affinity agonists for nicotinic acetylcholine receptors (nAChRs) [12,13] and β-adrenergic receptors (β-ARs) [14] have prompted investigations into the potential role of these neurotransmitter receptor families in smoking associated lung cancer initiation, progression and response to therapy. In light of the central role of these receptors and their respective neurotransmitters in the autonomic nervous system [15] as well as in the regulation of the mood [16] and psychological stress responses [17], these findings prompted recent experiments on the potential NSCLC promoting effects of psychological stress [18]. In turn, findings generated by these experiments lead to the hypothesis that neuropsychological factors may significantly impact lung cancer in smokers and non- smokers [19,20].

## 2. Functions of nAChRs and ARs

The families of nAChRs and ARs are expressed in the cell membrane of all mammalian cells. They serve as the recipients of neurotransmitters that are ligands for these receptors and are emitted from the autonomic nervous system, the brain, and the adrenal gland and from epithelial cells and the cancers arising from them [16,21].

Receptors comprising the nAChR family are ligand-gated ion channels enclosed by α subunits (α1–α10) in the presence (heteromeric nACHRs) or absence (homomeric nAChRs) of a variety of non-α subunits [22,23,24,25], with the α subunits serving as binding sites for agonists. The neurotransmitter acetylcholine is the physiological agonist for all nAChRs. Acetylcholine is synthesized and released by nerves of the vagus branch of the autonomic nervous system, by neurons in the brain and by numerous epithelial cells and the cancers arising from them [16,21]. Upon binding of an agonist to the nAChR α subunits, the ion channel opens, causing the influx of ions from the extra cellular environment into the cell, thereby depolarizing the cell membrane and triggering a host of cellular responses in a cell type-specific manner [16,24]. Of particular interest in the context of this review is the regulatory role of nAChRs for the release of neurotransmitters [26,27]. In turn, neurotransmitters released into the extra cellular environment bind as agonists to cell membrane receptors of the cells that release the neurotransmitters (autocrine) and adjacent (paracrine) cells. The release of the excitatory neurotransmitters norepinephrine, epinephrine, serotonin, dopamine and glutamate in the brain are regulated by the α7nAChR while the α4β2nAChR is primarily responsible for the regulation of the inhibitory neurotransmitter γ-aminobutyric acid (GABA) in the brain [26,28]. The neurotransmitters synthesized and released from epithelial cells and the cancers arising from them are similarly regulated, with the α7nAChR regulating serotonin [29,30] and jointly with the α3- and α5nAChRs epinephrine and norepinephrine [31,32,33] while the α4β2nAChR regulates GABA [33,34]. The expression of nAChRs with α subunits α1–α9 has been reported in normal airway and alveolar epithelium [16]. However, a comprehensive analysis of the expression and function of nAChR types in different histological types of lung cancer, including NSCLC, has not been conducted to date. Instead, the majority of preclinical investigations have focused on the role of the α7nAChR in the regulation of lung cancer proliferation, metastatic potential, resistance to therapy and angiogenesis (reviewed in [19,21,35,36]). While most of these studies have interpreted the observed activation of intracellular signaling pathways as direct responses to α7nAChR activation by agonist, *in vitro* experiments with small airway epithelial cells [32], NSCLC cells [34], colon cancer cells [31] and pancreatic ductal adenocarcinoma cells and pancreatic duct epithelial cells [33,37] have established that these cancer-stimulating responses were caused by the α7nAChR-mediated synthesis and release of the stress neurotransmitters norepinephrine and epinephrine which activated multiple signaling cascades downstream of β-adrenergic receptors in an autocrine fashion. In accord with the concept that nAChRs are redundant [23,24,27], it has also been shown that nAChRs with subunits α3 and α5 contribute to the stress-neurotransmitter-induced proliferation and migration induced by nicotine in epithelial cancer cells [33]. Moroever, it has been shown that small airway epithelial cells, NSCLC cells and pancreatic duct epithelial cells and the cancers derived from them express both isozymes of glutamate decarboxylase (GAD65, GAD67) that mediate the synthesis of the inhibitory neurotransmitter γ-aminobutyric acid (GABA) from glutamate and that they release GABA into the extracelullar environment [32,33,34].

Excitatory neurotransmitters, including norepinephrine and epinephrine, are important mediators of cognition, alertness and aggression in the brain while GABA conveys relaxation, contentment and happiness. Nicotinic receptors in the autonomic nervous system and in the adrenal gland additionally play important roles in the regulation of responses to psychological stress. The release of the stress neurotransmitters norepinephrine and epinephrine from sympathetic nerves is thus regulated by the α7 nAChR and by nAChRs with subunits α3 and α5 in the adrenal gland. Epinephrine and norepinephrine *in vitro* stimulate cell proliferation and migration of cancer of the breast [38], colon [31,39], prostate [40], pancreatic ductal adenocarcinoma and pancreatic duct epithelia [33] and of lung adenocarcinoma and small airway epithelia [32,34]. By contrast, GABA *in vitro* has been shown to inhibit cell proliferation and migration of cancer of the breast [38], colon [41], pancreas [37,42] and lung adenocarcinoma [34,43].

Nicotine binds as a selective agonist to all nAChRs with a significantly higher affinity than acetylcholine. However, nicotine does not bind with equal affinity to all nAChRs, demonstrating the highest affinity for nAChRs with the α4 subunit and the lowest affinity for the homomeric α7nAChR [22]. Accordingly, relatively high concentrations of nicotine are required to activate biological functions regulated by the α7nAChR whereas comparatively low nicotine concentrations still activate responses regulated by the α4nAChR. Like nicotine, the nicotine-derived carcinogenic nitrosamine N-nitrosonornicotine (NNN) is a preferential agonist for the α4nAChR, but its affinity for this receptor is about 5100× higher than that of nicotine [12,13]. By contrast, the nicotine-derived carcinogenic nitrosamine 4-(methylnitrosamino)-1-(3-pyridyl)-1-butanone (NNK) is a preferential agonist for the α7nAChR to which it binds with approximately 1300× higher affinity than nicotine [12,13]. It has been shown that similar to nAChR responses to chronic nicotine in the brain associated with nicotine addiction, chronic exposure of small airway epithelial cells *in vitro* to NNK upregulates the protein expression of the α7nAChR and sensitized the receptor, resulting in increased release of norepinephrine and epinephrine in response to lower concentrations of NNK whereas chronic NNK desensitized the α4nAChR, suppressing GABA release [32]. Nicotine has been used extensively as a pharmacological tool to study the pathobiology of nAChRs as it relates to nicotine addiction and cancer. However, many of the adverse effects of smoking traditionally blamed on nicotine may in reality be caused by NNK and NNN contained in tobacco smoke and formed additionally in the mammalian body from nicotine [44], with NNK concentrations up to 161 ng/mL reported in the pancreatic juice of smokers [45]. However, experimental behavioral studies to identify the potential contribution of NNK or NNN to the addictive effects of tobacco have not been conducted to date. The family of adrenergic receptors is comprised of two α-adrenergic receptors (α1-AR, α2-AR) and three β-adrenergic receptors (β1-AR, β2-AR, β3-AR). The β3-AR is exclusively expressed in adipose tissue whereas the remaining β-ARs as well as both α-ARs are expressed in most non-adipose tissues. All ARs are heptahelical transmembrane receptors coupled to G-proteins [46], but individual AR types are coupled to different G-proteins that activate different downstream effectors [19]. Cardiovascular functions are predominantly regulated by β1-ARs that are coupled to the stimulatory G-protein G_s_, which activates adenylyl cyclase/cAMP. Broad-spectrum β-AR antagonists (β-blockers as well as selective antagonists of the β1-AR (β1-blockers) are therefore widely used for the long-term management of cardiovascular disease [47]. On the other hand, β2-ARs regulate bronchodilation and agonists for these receptors are the active ingredient of inhalers used for the management of chronic obstructive pulmonary disease (COPD) and asthma [48]. The neurotransmitters norepinephrine and epinephrine are the physiological agonists for all ARs, with norepinephrine binding preferentially to the α-ARs and the β1-AR and epinephrine binding preferentially to β-ARs, with a higher affinity to β2-ARs than β1-ARs [49,50]. Both neurotransmitters are synthesized and released by nerves of the sympathicus branch of the autonomic nervous system, by neurons in the brain, by the adrenal medulla and by numerous epithelial cells and the cancers derived from them. Regardless of their anatomical location, their synthesis and release is regulated by nAChRs [21,50]. Because norepinephrine and epinephrine released from sympathicus nerves and the adrenal medulla mediate responses of the mammalian body to psychological stress, these neurotransmitters are commonly referred to as “stress neurotransmitters”. Intracellular increase in cAMP and activated protein kinase A (PKA) downstream of β-ARs additionally regulate the synthesis and release of the epidermal growth factor (EGF) [51], amphiregulin [52] vascular endothelial growth factor (VEGF) [53] and arachidonic acid (AA) [14], all of which are important mediators of malignant potential in numerous cancers, including NSCLC.

The nicotine-derived carcinogenic nitrosamine NNK is an agonist for β-ARs with a 600× greater affinity than norepinephrine to the β1-AR and a 2200× greater affinity for the β2-AR than epinephrine whereas NNN does not bind to these receptors [14]. *In vitro* studies have shown that β-AR agonists, such as norepinephrine, epinephrine, isoproterenol or NNK stimulate the proliferation and migration of cancer of the mammary gland [38], colon [31,39], prostate [40], pancreas [54,55] and adenocarcinoma of the lung [14,32].

## 3. Impact of “Nicotine Addiction” on Smoking Associated Lung Cancer

**Figure 1:**
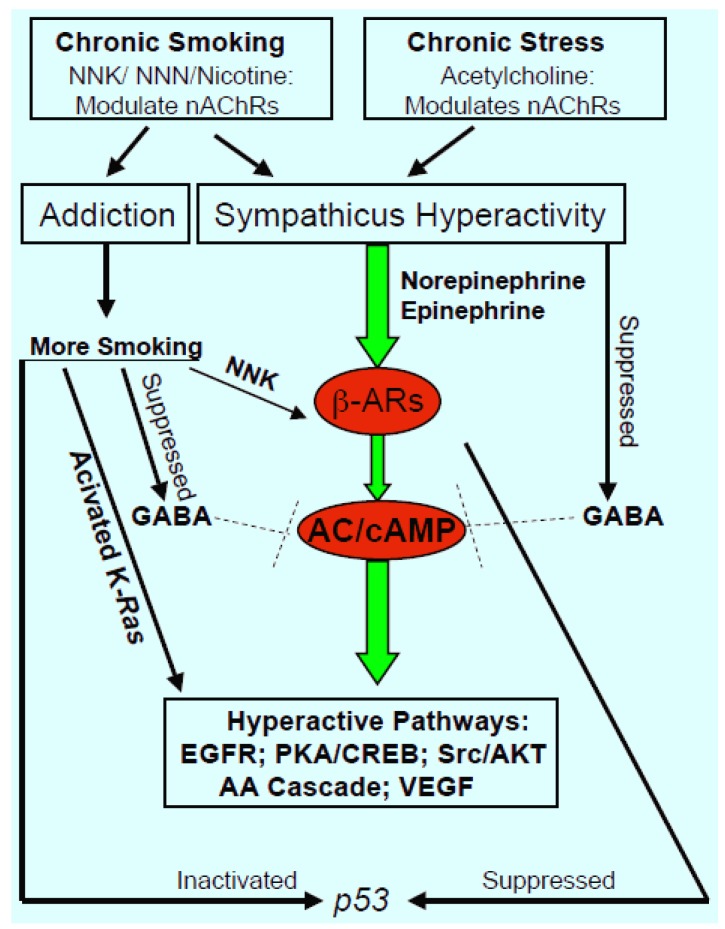
Working model illustrating the central role of sympathicus hyperactivity caused by chronic smoking and chronic psychological stress in the regulation of smoking-associated lung adenocarcinoma.

Reactive metabolites of the nicotine-derived carcinogenic nitrosamines NNN and NNK interact with the DNA molecule to form activating point mutations of K-*Ras* and inactivating mutations of *p53* [11]. Activating point mutations in K-*Ras* represent the most common oncogenic alteration in lung adenocarcinoma [56]. *Ras* is an important signaling protein in numerous pathways that regulate cell proliferation and migration, including the EGFR pathway, the Src/AKT pathway and the AA cascade, all of which are activated when endogenous or exogenous agonists bind to β-ARs (Figure 1). On the other hand, the tumor suppressor gene *p53* regulates the induction of apoptosis, resulting in a lack of responsiveness to apoptosis-inducing agents, such as cancer therapeutics, upon its mutational inactivation.

“Nicotine addiction”, which due to the binding kinetics of NNN and NNK to nAChRs (see above) may be caused to a significant extent by these nicotine derivatives, is characterized by post-transcriptional and post-translational upregulation in the protein expression of all nAChRs [57]. This change in receptor protein is accompanied by sensitization to agonist of the α7nAChR whereas the α4β2nAChR is desensitized [22,28,58]. With the α7nAChR regulating excitatory neurotransmitters, including norepinephrine and epinephrine, and the α4β2nAChR regulating the inhibitory neurotransmitter GABA, excitatory neurotransmitters predominate and GABA is suppressed, resulting in the tension and craving characteristic for addiction when these nAChR changes occur in the brain. In response, smokers increase their daily consumption of cigarettes, thus increasing their exposure to carcinogenic tobacco components that enhance their risk for smoking-associated cancer, including lung cancer (Figure 1).

Due to the fact that nicotine as well as NNN and NNK are distributed throughout the entire body, the adaptive changes of nAChRs in response to chronic tobacco-specific agonists are not limited to the brain but are ubiquitously occurring in all nAChRs of the mammalian body. In the autonomic nervous system, the adaptive upregulation and sensitization of the α7nAChR increases the release of norepineprhine and epinephrine from nerves of the sympathicus, resulting in sympathicus hyperactivity.

Sympathicus hyperactivity has been recognized as an important mediator of cardiovascular disease [59,60,61,62], the most common non-neoplastic disease associated with smoking. In addition, the increased blood levels of norepinephrine and epinephrine caused by sympathicus hyperactivity provide a selective growth advantage for cancers regulated by β-ARs, including the most common human lung cancer, adenocarcinoma. *In vitro* studies have shown that chronic exposure to nicotine or NNK also upregulates and sensitizes the α7nAChR expressed in small airway epithelial cells [32], pancreatic duct epithelial cells and pancreatic cancer [33,37], thereby increasing the production of norepinephrine and epinephrine, which are autocrine growth factors for these cells. This cancer promoting effect is further enhanced by the simultaneous suppression of cancer inhibitory GABA production due to desensitization of the α4β2nAChR in these cells [32,37,63]. As is shown in Figure 1, hyperactive β-AR signaling in response to systemic and autocrine increases in their physiological agonists thus further intensifies the cancer-stimulating signaling pathways that are additionally activated by the presence of constitutively active K-*Ras*.

Numerous studies *in vitro* and in mouse xenografts [21,64,65,66] with nicotine doses comparable to serum levels in smokers have reported significant promoting effects on cell proliferation, migration and metastasis. These studies have provided valuable information on the role of nAChRs in cancer progression but do not necessarily prove that the nicotine in tobacco products promotes smoking-associated cancer. Due to the much higher affinities of NNK and NNN to the α7nAChR and α4β2nAChR, respectively, nicotine will be displaced from these receptors by the two nitrosamines unless present at a 5100× higher concentration than NNN and at a 1300× higher concentration than NNK. The situation is different in nicotine replacement products that contain significantly lower concentrations of nicotine and only yield negligible levels of nitrosamines formed endogenously from nicotine [67]. Comparative investigations with xenografts from identical pancreatic cancer cell lines have shown that only the high nicotine doses comparable to serum levels in smokers increased xenograft growth [68] whereas low nicotine comparable to NRT products did not [69]. By contrast, the low nicotine treatments significantly increased resistance of the xenografts to the cancer therapeutic gemcitabine by inhibiting apoptotic pathways [69]. Similar dose-dependent differences of nicotine have been reported in lung cancer cell lines *in vitro*, with 1 μM nicotine stimulating cell proliferation [29,70] and 200 nM nicotine inhibiting drug-induced apoptosis while failing to increase cell proliferation [71]. The lack of proliferation in response to low dose nicotine is caused by the low affinity of nicotine to the α7nAChR that regulates cell proliferation. In accord with these observations, recent publications from two independent laboratories have reported that low dose nicotine comparable to levels yielded with NRT products did not promote lung cancer development, proliferation and metastasis in mice treated with a high dosing regimen of NNK that by itself yielded a 100% lung tumor incidence [72,73]. It would seem desirable to conduct studies of this nature with a dosing regimen of carcinogen that yields a significantly lower than 100% tumor incidence so that significant promoting effects become actually detectable. Nevertheless the failure of low dose nicotine to induce cancer cell proliferation reported by the two above cited independent laboratories [69,71] in conjunction with the very high affinity of NNK for the α7nAChR render it unlikely that NRT level nicotine would promote NNK-induced lung cancer in smokers. However, the reported increase in resistance of cancer cells to therapeutics by low dose nicotine remains a serious concern [69,71] and non-nicotine products should be used instead of nicotine for nicotine replacement therapy in individuals undergoing cancer therapy.

## 4. Impact of Chronic Psychological Stress on Lung Cancer

It has been reported that people often smoke to reduce psychological stress [74,75], that individuals with anxiety disorders are over-represented among smokers [76] and that smoking is disproportionately prevalent in individuals with posttraumatic stress disorder [77]. The responses of the mammalian body to psychological stress are mediated by the release of the stress neurotransmitters norepinephrine and epinephrine from the adrenal gland, from the sympathicus branch of the autonomic nervous system and by the release of the stress hormone cortisol from the adrenal gland [17]. In turn the release of norepinephrine and epinephrine from sympathicus nerves is regulated by the homomeric α7nAChR [78] while heteromeric nAChRs containing the subunits α3 and α5 regulate their release from the adrenal gland [79]. As explained earlier in this review, smoking has similar effects on stress neurotransmitters caused by interaction of the same nAChRs with nicotine. Similar to smoking [80,81], chronic stress also suppresses the GABA system [82]. The stress-induced systemic predominance of stress neurotransmitters stimulates the growth of cancers that express β-adrenergic receptor–mediated regulatory pathways (Figure 1) [19,83] and individuals who smoke to cope with stress further exacerbate their already existing sympathicus hyperactivity. Adenocarcinoma, the most common lung cancer in smokers and non-smokers, expresses this pathway and its proliferation and migration are significantly increased *in vitro* by β-AR agonists that activate multiple signaling cascades, including the EGFR pathway, the AA cascade, protein kinase A/CREB, Src/AKT signaling and others [14,32,84,85]. Treatment of hamsters with epinephrine significantly promoted NNK-induced lung adenocarcinoma development whereas the β-blocker propranolol had significant preventive effects [86]. Experimentally induced chronic social stress significantly increased the growth of mouse xenografts from lung adenocarcinomas with and without activating mutations in K-*Ras* [18]. The tumor-promoting effects of stress were associated with increased systemic and tumor levels of stress neurotransmitters and cAMP and suppression of GABA, while the protein expression of nAChR subunits α3, α4, α5, and α7 and phosphorylated signaling proteins CREB and ERK was increased in xenograft tissues. All of the described adverse effects of social stress were completely prevented by simultaneous treatment of the mice with GABA in the drinking water [18]. *In vitro* studies with human adenocarcinoma cell lines suggest that the observed strong preventive effects of GABA on lung adenocarcinomas were mediated by the inhibition of cAMP formation due to inactivation of adenylyl cyclase, the enzyme activated by the stimulatory G-protein G_s_ of β-ARs [43]. Experimentally induced psychological stress also enhanced the growth of pancreatic cancer xenografts [87] and epinephrine increased the metastatic potential of ovarian cancer cells [88]. In addition, it has been shown that chronic treatment with epinephrine or chronic restraint stress suppressed the tumor suppressor gene *p53* via β2-AR signaling in mice and that these responses were abolished by the β-blocker propranolol [89,90]. The importance of hyperactive β-adrenergic receptor signaling in the promotion of NSCLC development and progression is further underlined by a recent report that NSCLC patients undergoing radiation therapy had improved survival outcomes with incidental use of β-blockers [91]. β-Blocker use has also improved relapse-free survival in breast cancer patients [92].

## 5. Conclusions

The mutational activities of metabolites formed from tobacco carcinogens are generally considered key events for the initiation of smoking-associated lung cancer. However, racial disparities [7], the prevalence of adenocarcinoma in women [4] and the reported association of psychological distress with increased lung cancer mortality [93] suggest the involvement of other factors in addition to smoking. Experiments in laboratory animals with a lifespan of only two to three years typically employ very high doses of tobacco carcinogens aimed at approaching carcinogen amounts similar to the lifetime exposure in smokers. It is therefore not surprising that the widely used mouse model of NNK-induced lung carcinogenesis responds with a 100% lung cancer incidence to NNK treatment [72,73], as opposed to a lung cancer incidence of up to 28% in heavy smokers [94] with an average life expectancy of 70 years. While such animal models are valuable tools for the identification of carcinogens, cancer preventive and therapeutic agents, they may not provide optimum conditions for mechanistic studies of cancer initiation, promotion and progression. The data and interpretations summarized in this review support the hypothesis that neuropsychological factors play key roles in establishing a biological environment that permits cancer cells to develop and progress. The ability of the mammalian organism to survive throughout evolution is mediated by the autonomic nervous system that governs adaptive responses to endogenous changes as well as external and emotional signals. Adaptive changes in the function of nAChRs in response to chronic exposure to nAChR agonists in tobacco smoke (NNK, NNN, nicotine) or released in response to chronic psychological stress (acetylcholine) destroy the ability of the autonomic nervous system to maintain homeostasis. The resulting sympathicus hyperactivity with excessive release of stress neurotransmitters greatly enhances via β-adrenergic receptor signaling the activity of multiple cellular pathways known to stimulate the proliferation, migration, angiogenesis and metastasis of NSCLC (Figure 1). These effects are further intensified by the simultaneous suppression of the inhibitory GABA system and the β-AR-mediated suppression of p53. Cellular responses to the mutational changes in K-*Ras* and *p53* caused by tobacco carcinogens therefore remain unchecked and can no longer be compensated for by corrective signals from the autonomic nervous system. Estrogen additionally intensifies the signals downstream of β-ARs by transactivating the β1-AR [95], a phenomenon that may contribute to the predominance of lung adenocarcinoma in women [4]. On the other hand, the prevalence of low socio-economic status and associated chronic psychological stress in African Americans may contribute to the racial lung cancer disparities reported [7]. In addition, β-adrenergic signaling is enhanced by broncho-dilating drugs that either act as β2-AR agonists or increase cAMP signaling by inhibiting phosphodiesterases. The long-term use of these agents may thus significantly contribute to the increased lung cancer risk observed in individuals with chronic obstructive pulmonary disease (COPD) [96]. Efforts to target individual signaling proteins in combination with chemo- or radiation therapy have not significantly improved the prognosis of NSCLC [1]. Additional approaches aimed at normalizing sympathicus hyperactivity are needed to improve clinical outcomes.
